# Self-esteem consistency predicts the course of therapy in depressed patients

**DOI:** 10.1371/journal.pone.0199957

**Published:** 2018-07-25

**Authors:** Carolin Eberl, Isabell Winkler, Steffen Pawelczack, Eva Tröbitz, Mike Rinck, Eni S. Becker, Johannes Lindenmeyer

**Affiliations:** 1 Behavioural Science Institute, Radboud University Nijmegen, Nijmegen, The Netherlands; 2 Department of Psychology, Chemnitz University of Technology, Chemnitz, Germany; 3 Salus Clinic Lindow, Lindow, Germany; 4 Institute for Psychology, University of Leipzig, Leipzig, Germany; 5 Faculty of Psychology, Ruhr-University Bochum, Bochum, Germany; Istituto Superiore Di Sanita, ITALY

## Abstract

Previous studies on self-esteem and depression demonstrated the usefulness of both implicit and explicit self-esteem as well as their congruence (also known as self-esteem consistency) to predict future depressive symptoms. High self-esteem consistency describes when implicit and explicit self-esteem match (e.g., both high or both low). In the current study, we investigated if implicit and explicit self-esteem and self-esteem consistency predict the course of treatment efficacy of a cognitive behavioral depression therapy. Explicit self-esteem was assessed by the Rosenberg Self-Esteem Scale, implicit self-esteem by a priming task. Participants were 31 patients with a major depressive or recurrent depressive disorder receiving cognitive behavioral therapy treatment in an inpatient setting. Self-esteem measures were administered before treatment. The development of depression symptoms during treatment and at the 4-month follow-up was measured on the Beck Depression Inventory. Implicit and explicit self-esteem did not predict the course of the therapy. Patients with congruent self-esteem, however, improved faster and showed lower severity of symptoms throughout treatment. In contrast, neither explicit nor implicit self-esteem nor self-esteem consistency predicted the stability of effects after treatment. Practical implications such as targeting discrepancies in self-esteem during treatment are discussed.

## Introduction

Depression is the most disabling type of mental disorders, and therefore, as primary and secondary diagnosis it is one of the most frequent reasons for starting a treatment in psychotherapy [1]. In the scope of depression treatment, cognitive behavioral therapy (CBT) is an important kind of therapy with a generally satisfying treatment success. However, CBT is not always the most effective and efficient treatment and it does not have lasting effects for all types of patients [2]. A better understanding in which cases CBT–potentially in combination with other forms of therapy–could acquire an enhanced efficacy may be gained from research on self-evaluation. Negative self-evaluations are thought to be core symptoms of depression. Especially cognitive theories of depression propose that depressed individuals process self-relevant information in a dysfunctional and negative manner [3]. From the recent literature it is known that not only explicit, but also implicit cognitions are relevant to the understanding of depression, and it is necessary to differentiate between explicit and implicit self-evaluation [4]. Implicit cognitions have been defined as automatic processes [5], which are difficult to control, and occur unintentionally and effortlessly [6]. Explicit cognitions, on the other hand, are rational and rather sophisticated judgments available to conscious introspection. Dual-process models like the one of Strack and Deutsch [7] postulate that explicit and implicit processes build two independent, but interacting systems: the reflective and the impulsive system. Both systems run in parallel and result in a common pathway: the activation of behavioral schemata. The reflective system (based on explicit processes) requires a lot of cognitive capacity, and thus, is more likely to control behavior when respective resources are available. In these situations, behavior will mostly be rational. The impulsive system (based on implicit processes), however, can work sufficiently well even under conditions of very high mental load, and is therefore more likely to control behavior when basic needs are deprived and when cognitive capacity is low (e.g., in stressful life situations or depressive moods). The interaction of both systems can explain the development and maintenance of depressive disorders [8]. Implicit processing is used to maintain mood by retrieving mood congruent information whereas reflective processing is used to change mood when a certain affect intensity or duration is reached. Reflective processing then retrieves mood incongruent information in order to modify current mood state. However, when cognitive resources are low, when negative biases do not violate expectancies, and when reflective processing cannot adjust a negative bias, the implicit processing will not be corrected. This can be a starting point of a depressive downward spiral. Patients with a more sever recurrent depression disorder showed stronger associations between the self and negative descriptors than patients with less severe depression [9]. Individuals with a remitted depression disorder maintained these associations [10]. According to Beevers [8], uncorrected negatively biases can lead to dysphoric mood that becomes persistent and deplete cognitive resources, which in turn, reinforces negatively biased implicit processing. Over time, a feedback loop can build between implicit processing and dysphoric mood that increases the probability of the development of depressive episodes. Thus, the individual characteristics of both explicit as well as implicit cognitions have to be taken into account in order to correctly predict the development and maintenance of depressive disorders.

In what follows, we will focus on the relationship between self-esteem and depression, and thereby differentiate between *explicit self-esteem (ESE)*, which is the conscious and deliberately reasoned evaluation of the self, and *implicit self-esteem (ISE)*, an automatic, intuitive evaluation of the self that guides spontaneous reactions to self-relevant stimuli [11]. The advantages of measuring ISE by means of priming tasks are that it is not confounded with social desirability, it cannot be deliberately influenced as easily as self-rating questionnaires (the usual way ESE is measured), and it does not depend on the ability of self-reflection. Therefore, it may give access to more spontaneous self-evaluations [11]. Greenwald and Farnham [12] showed that in general, people hold a positive implicit attitude towards themselves (positivity bias), while ESE and ISE are only weakly correlated (see also [11,13]). Furthermore, whereas previous studies consistently showed that ESE has a strong inverse relationship with depression [14] and suicidal ideation [15], the relationship between ISE and depression is more ambiguous. De Raedt et al. [16] studied ISE in depressed inpatients and found that patients and healthy controls did not differ in ISE, both showing a positivity bias. This was replicated by Franck et al. [17] who compared currently depressed individuals to formerly and never-depressed ones. Taken together, the results support the idea of different underlying learning mechanisms of ESE and ISE. Whereas explicit learning is a result of conscious evaluations, implicit learning is rather governed by automatic mechanisms such as classical conditioning [18]. ISE is thought to develop earlier than ESE and stems, at least partly, from early social interactions [19], while ESE is assumed to consist of more sophisticated cognitive judgments of the self [20]. Concerning self-esteem, a person could therefore hold two independent attitudes towards himself or herself, for instance positive ESE, which may be due to success and deliberate evaluations of achieved life goals, and negative ISE, which may result from experienced nonverbal hostility in social situations. It can be assumed that ESE and ISE are rather stabile cognitions, which can affect each other mutually, at least to some extent, and may be modified by new information. Since both kinds of cognitions are the results of different learning mechanisms, distinct techniques are required for their modification. Cognitive behavioral therapy addresses mainly explicit cognitions. It targets explicit motivations, discusses decisions, and plans future coping behavior. In contrast, the impulsive system will have to be addressed by changing implicit processes. Cognitive bias modification, for example, achieves this aim by inducing or reducing cognitive biases by means of classical and instrumental conditioning. Taken together, both types of self-esteem have to be addressed in an adequate way to enable a successful therapy.

In recent years, research has focused on the interaction of ESE and ISE. It is possible to differentiate between *congruent* self-esteem, which implies a fit of explicit and implicit self-esteem (e.g., both high or both low), and *discrepant* self-esteem, in which low ESE and high ISE are combined (so-called "damaged self-esteem") or high ESE and low ISE (so-called "fragile self-esteem") [21–23]. Schröder-Abé et al. [24] showed that compared to congruent self-esteem, both types of discrepant self-esteem were related to more anger suppression, more depressive attributional style, more nervousness, and more days of impaired health. Interestingly, both congruent combinations were related to fewer health problems than the two discrepant self-esteem combinations. This suggests that high ESE or high ISE is not always advantageous in protecting the self against outer threat, and it demonstrates the disadvantage of holding discrepant self-esteem. Briñol et al. [25] showed that in order to resolve the negative, unpleasant, or dysfunctional consequences that often accompany discrepant self-conceptions, persons more strongly engage in examining information relevant to the discrepancy. Similarly, Phillips and Hine [26] found that depressive rumination (seen as an attempt to detect ways to reduce depressive symptoms while depleting cognitive resources and amplifying negative implicit biases) occurred more frequently in participants holding a damaged self-esteem. Obviously, holding a discrepant self-esteem is a risk factor in terms of psychological health, which could be reduced by an adequate therapy.

Unfortunately, very few studies examined the combination of ISE and ESE in depression. An exception was reported by Franck et al. [27], demonstrating that currently depressed patients with suicidal ideations possessed high discrepancies in their self-esteem (high ISE combined with low ESE, i.e., damaged self-esteem), whereas currently depressed individuals without suicidal ideations possessed congruently low self-esteem. In a matched non-clinical control group ISE and ESE were congruent, and in comparison to depressed patients without suicidal ideations significantly higher regarding both types of self-esteem. Creemers et al. [28] examined the effects of extent and direction of the discrepancy between ISE and ESE on suicidal ideation, depressive symptoms, and loneliness in a student sample, and found that the extent of discrepancy had an impact on suicidal ideation as well as depressive symptoms only when ISE was higher than ESE (damaged self-esteem). In a recent study by van Tuijl et al. [29] the discrepancy between ISE and ESE was not associated with disorder status (current major depressive disorder versus never depressed controls) after controlling for ESE. ESE was higher in healthy controls compared to patients with a current, remitted, and recovered major depressive disorder. In this study, in contrast to Creemers et al. [28], only the diagnostic status and not the extent of depressive symptoms was used as dependent variable. Therefore, it is still an open question how extent and direction of the discrepancy between ISE and ESE affect the intensity of depression and the therapy process.

In the current study, we investigated whether discrepant self-esteem in depressed patients is related to severity of symptoms, disadvantages concerning the course of therapy and stability of treatment effects. We formulated the following hypotheses: In depressed patients, (1) discrepant self-esteem is related to more severe symptoms than congruent self-esteem, (2) discrepant self-esteem is related to a slower symptom reduction during treatment than congruent self-esteem, and (3) discrepant self-esteem is related to less stability of treatment effects than congruent self-esteem. Also, in an explanatory way, the effects of the direction of self-esteem discrepancy were examined.

## Methods

### Participants

Participants were 9 male and 22 female depressed patients (with a mean age of 46.9 years; *SD* = 8.3) receiving inpatient treatment at the Salus Clinic in Lindow, Germany, a rehabilitation clinic to which patients were assigned by the German Pension Fund. Patients could participate in the study if depression was their main treatment target and if they took part in group therapy. Inclusion criteria were a diagnosis of a major depressive disorder (*n* = 14, ICD-10: F32), which means patients were in an acute depressive episode without having experienced any former depressive episodes, or a diagnosis of Recurrent Depressive Disorder (*n* = 17, ICD-10: F33), which means they were in an acute depressive episode and had experienced at least one former depressive episode. The diagnoses were classified by means of the International Statistical Classification of Diseases and Related Health Problems (ICD-10; [30]), and assessed by professionals using a computerized version of the Composite International Diagnostic Interview (CIDI; [31]), which assists in checking and classifying symptoms. The diagnostic interview was complemented by questionnaires such as the Beck Depression Inventory (BDI; [32]; German version by Hautzinger et al. [33]) and the Symptom Checklist (SCL-90-R; [34]; German version by Franke [35]). Both the CIDI and the questionnaires were the basis for the final expert ratings on diagnoses, made by clinical psychologists. Moreover, patients had to have a minimum score of 18 on the BDI, which is considered to be the cut-off for clinical relevance [33]. For patients suffering from a major depressive disorder, the mean BDI score was 24.0 (*SD* = 4.8), for patients with a recurrent depressive disorder, it was 27.9 (*SD* = 6.8). Exclusion criteria were cognitive or verbal impairments and not being a native German speaker.

The average duration of the depressive disorder for patients suffering from a major depressive disorder, that is, the duration of the current depressive episode, was 2.3 years (*SD* = 4.6), according to the information given by the patients in the diagnostic interview. The mean period since the occurrence of their first depressive episode for patients suffering from a recurrent depressive disorder was 9.4 years (*SD* = 7.1), indicating rather chronic cases of depression. Of the 31 patients, 14 had more than one diagnosis. The most frequent comorbid diagnosis was nicotine dependence (F17.2; *n* = 10), followed by other substance use disorders, mainly alcohol abuse (F10.1; *n* = 5) and alcohol dependence with current abstinence (F10.21; *n* = 7). Two patients suffered from pain disorders and another two from personality disorders. Suicidal ideation was reported by 18 of the patients. Medication was not an exclusion criterion. Twelve patients were without psychiatric medication, 18 took antidepressants. Out of the 18, one took antidepressants in combination with antipsychotics and one in combination with sedatives. One patient used sedatives only. For some of these patients the additional drug therapy started in the clinic, for others the medication started before the beginning of the inpatient treatment. The duration of the therapy differed depending on the progress of the patients concerning the symptom reduction. It ranged from four to eight weeks with a mean of 5.4 weeks (*SD* = 1.2). Patients gave their written informed consent for participating in the study. The study was approved by the ethics committee of the German Pension Fund.

### Procedure

During the first week of the diagnostic phase, participants took part in the pretest session as part of the clinic’s entrance routine. Pretesting of ISE and completing some questionnaires (including a scale for measuring ESE) was part of the clinic’s entrance procedure, so no additional materials had to be administered for the study. Participants were informed about the study and gave their informed consent. During the test session, participants completed a battery of computer tasks, which took about 90 minutes. Along with other measures not relevant to the present study, participants completed a semantic priming task very similar to the one used by Hetts et al. [36] as the measure of ISE, and they completed the Rosenberg Self-Esteem Scale (RSES; [37]; revised German version by von Collani & Herzberg [38]) as the measure of ESE. Headphones prevented mutual disruption during group testing. A research assistant was present at all times to answer questions. Note that the participants of this study did not participate in any other study conducted at the clinic.

All patients received a daily group therapy session and individual sessions focusing on symptoms and special treatment of depression according to Hautzinger [39]. Patients completed the BDI at the beginning of their therapy (BDI-1) and at its end (BDI-5), as well as once a week while they attended the group sessions (BDI-2, BDI-3, BDI-4). Since the duration of the therapy differed between patients, the period between BDI-4 and BDI-5 was not constant. Additionally, the patients completed the BDI at the 4-month follow-up (BDI-6). For that, all participants were called by phone during a one-week period at about four months after the end of treatment.

### Instruments and measures

The **Beck Depression Inventory** (BDI; [32]; German version by Hautzinger et al. [33]) was used to measure the severity of the depressive disorder. Internal consistency (α = .80) and test-retest reliability (*r* = .92) are high [40].

The **Rosenberg Self-Esteem Scale** (RSES; [37]; revised German version by von Collani & Herzberg [38]) is a 10-item self-report scale that addresses feelings of global self-worth, and thus, measures ESE. All responses are given on a scale ranging from 1 (strongly disagree) to 6 (strongly agree). The internal consistency of the scale is high (α = .85), and it shows a reasonable test-retest reliability of *r* = .87 [11].

A **Priming Task** [36] was conducted to measure ISE. When primed with certain concepts, such as the self or others, people are able to identify or categorize target words more quickly that are highly associated with the prime than non-associated target words. As the self-relevant prime, we used the word “ich” (German for "I"), the other-relevant prime was the word "andere" (German for “others”). On each trial of the priming task, after being exposed to either one of the two primes, participants had to categorize a target word appearing in the middle of the screen into pleasant vs. unpleasant. They responded by pressing one of two marked keys on the right vs. left side of the keyboard. Primes were presented at random. The target words were words that could be used to evaluate individuals. English translations of the positive and negative target words are, respectively: winner, smart, precious, strong, competent, good, successful, popular; versus loser, unsuccessful, wimp, inferior, bad, weak, dumb, worthless. The priming task consisted of 212 trials in total. During each trial, participants first saw a fixation cross in the middle of the screen for 1,000ms. After a pause of 500ms, the prime word was displayed for 300ms. Subsequently, the target word was presented until participants pressed one of the two keys. If they responded incorrectly or failed to press a key within 5 seconds, an "Error" message was displayed. There were 20 practice trials, followed by two blocks of 96 test trials each. The two prime conditions (self, others) were equally often followed by positive vs. negative targets.

For computing ISE scores, four composite scores were first computed for each participant, after excluding outlier reaction times (RT)–the fastest and slowest 1%: (a) mean RT for positive targets preceded by self-related primes, (b) mean RT for negative targets preceded by self-related primes, (c) mean RT for positive targets preceded by other-related primes, and (d) mean RT for negative targets preceded by other-related primes. From these four mean RTs, an overall priming score was computed for each participant, according to the formula (b+c)-(a+d). Positive values of the priming score indicate positive ISE. Finally, after the examination of the distribution of the ISE-values extreme outliers were capped by limiting the ISE-range between -200ms and 200ms, thereby two ISE scores (one negative and one positive) were set to -200ms and 200ms, respectively.

### Statistical analysis

To investigate the development of the depression symptoms over the course of the therapy, we calculated a multilevel regression analysis using the software HLM7 [41]. Multilevel regression analysis has been proven very suitable for paradigms such as the one in the current study that include repeated measures at level 1—the different BDI measurements—and person variables at level 2—the self-esteem measures (see [42–44]). In this analysis, to calculate the “treatment-effect model” we used five BDI scores (BDI-1 to BDI-5) as the dependent variable, that is, all scores collected while the therapy took place, and the three self-esteem measures ESE, ISE, and the consistency of ESE and ISE (i.e., self-esteem discrepancy) as predictors. Self-esteem discrepancy was computed as the absolute difference of *z*-standardized ESE and ISE scores, which means that if ESE and ISE were congruent, e.g., both having comparable positive or negative *z* scores, a small difference was obtained, and if ESE and ISE were discrepant, for example, one markedly positive and one markedly negative *z* score, a large difference occurred. Additionally, the sign of the difference between ESE and ISE might be an important source of information. Therefore we included the dichotomized difference between the z scores of ESE and ISE as an additional predictor—coded 1 if ESE was larger than ISE and -1 otherwise.

We computed the diagnostic effects over all BDI scores included in the model. The higher the regression coefficients, the better the different self-esteem variables predicted the patients’ average BDI scores. In addition, we calculated the effects of the therapy process, that is, the development of the BDI scores over the course of the therapy. We used a linear prediction for the five points of measurement (weights of 1, 2, 3, 4, and 5). Here, a negative regression coefficient means decreasing BDI scores during the therapy. Moreover, we report the effects of the self-esteem measures on the therapy process (i.e., the interaction effect of each of the self-esteem measures and point of measurement). The higher the regression coefficients, the better the variables predicted the development of the BDI scores over the therapy process (e.g., a stronger reduction of the depression symptoms). All effects are reported as standardized regression coefficients. For the diagnostic effects, we expected negative regression coefficients for ESE and ISE (i.e., the higher ESE or ISE, the lower the average BDI scores), and positive regression coefficients for self-esteem discrepancy (i.e., the higher the self-esteem discrepancy, the higher the average BDI scores). For the therapy-process effects, we expected positive regression coefficients for ESE and ISE (i.e., the higher ESE or ISE, the stronger the expected reduction of the depressive symptoms over the course of the therapy) and negative regression coefficients for self-esteem discrepancy (i.e., the higher the self-esteem discrepancy, the lower the expected symptom reduction over the course of the therapy).

To examine the influence of the self-esteem measures on the stability of the treatment (the “treatment-stability model”), a simultaneous multiple regression analysis was calculated using the difference between the BDI scores at the 4-month follow-up (BDI-6) and at the end of the therapy (BDI-5) as the dependent variable and the three self-esteem measures ESE, ISE, and self-esteem discrepancy, as predictors. We expected a higher treatment stability and thus lower values for the difference between BDI-6 and BDI-5 for patients with higher ESE, higher ISE, and especially with lower self-esteem discrepancy. Here, the higher the (negative) regression coefficients, the better the different self-esteem variables predicted the differences between BDI-6 and BDI-5. Also in this analysis, we included the sign of the difference between ESE and ISE as an additional predictor to test whether the kind of possible self-esteem inconsistency (damaged or fragile self-esteem) plays a role in the treatment stability.

In both the multilevel analysis for examining the treatment-effect model and the simultaneous regression analysis for investigating the treatment-stability model, we controlled for the impact of patients’ gender, age, main diagnosis (i.e., major depressive or recurrent depressive disorder), treatment duration in weeks, the comorbid diagnosis of alcohol abuse (yes or no) and suicidal ideation (yes or no), since these variables can be expected to influence the degree of depressive symptoms as well as the treatment progress and treatment stability. This was done by additionally including the respective variables as predictors in the analyses. All assumptions for the calculation of the multilevel and the simultaneous regression analyses were checked before conducting the analyses with no obtained violation. Especially no or only little multicollinearity between the predictor variables was obtained.

## Results

Overall, the mean ISE score did not significantly differ from zero (*M* = -11.9, *SD* = 98.5), *t*(30) = 0.67, *p* = .508, *d* = 0.12, and ISE was not correlated with ESE (*r* = -.09, *p* = .650). The RSES mean score (ESE) in the present sample was 36.6 (SD = 9.9) with a scale range from 10 to 60. Although this seems to be a relatively high mean for a clinical sample of patients in a depressive episode, it is similar to the RSES mean score (of 26.1 (*SD* = 5.2) with a scale range from 10 to 40) in a comparable study including 60 patients with a current major depressive disorder [29]. ISE scores did not significantly differ between patients with a major depressive disorder (*M* = 24.5, *SD* = 90.7) and patients with a recurrent depressive disorder (*M* = 1.5, *SD* = 106.1), *t*(29) = 0.64, *p* = .527, *d* = 0.24, yet there was a significant difference in ESE between patients with a major depressive disorder (*M* = 42.1, *SD* = 10.4) and patients with a recurrent depressive disorder (*M* = 32.0, *SD* = 7.0), *t*(29) = 3.22, *p* = .003, *d* = 1.20. Of the 31 patients, 18 held a higher ESE than ISE, and for 13 the opposite was true.

Table 1 includes the descriptive statistics of all metric predictor and outcome variables. On average, the depressive symptoms reduced significantly from the beginning of the therapy (BDI-1) to its end (BDI-5): *t*(30) = 7.42, *p* < .001, *d* = 1.35. The symptom reduction was relatively stable four months after the therapy (BDI-6). There was still a significant difference in the degree of depressive symptoms between BDI-1 and BDI-6 (*t*(30) = 5.71, *p* < .001, *d* = 1.04) and no difference between BDI-5 and BDI-6 (*t*(30) = -1.22, *p* < .234, *d* = -0.22). Although there is a trend for the depressive symptoms to increase in the follow-up measurement, results did not reach statistical significance. Bivariate correlations among the primary predictor and outcome variables are displayed in Table 2. Self-esteem discrepancy correlated positively with the BDI-1 to BDI-5 scores, but not with BDI-6 score. The correlations between ESE as well as ISE and the BDI scores were markedly lower, with the exception of the correlation between ISE and the BDI-1 score. Additionally, suicidal ideation correlated positively with self-esteem discrepancy. Furthermore, the main diagnosis as well as the treatment duration correlated negatively with ESE, meaning that ESE was higher for patients with a Mayor Depression Disorder compared to patients with a Recurrent Depressive Disorder, and that patients holding a higher ESE received a shorter treatment duration.

**Table 1:** Descriptive statistics of all metric predictor and outcome variables.

Variables	Mean	SD	Min	Max
Age	46.9	8.27	27	63
ESE	36.6	9.92	20	60
ISE	11.9	98.5	-200	200
SE-discrepancy	1.18	0.85	0.09	3.36
Treatment duration	5.40	1.17	4	8
BDI-1	26.1	6.21	19	41
BDI-2	21.5	9.53	4	41
BDI-3	17.7	11.5	0	40
BDI-4	16.1	11.3	0	38
BDI-5	13.1	10.1	0	35
BDI-6	15.1	11.5	0	42

ESE = explicit self-esteem; ISE = implicit self-esteem; SE = self-esteem; BDI = Beck Depression Inventory; age in years; treatment duration in weeks.

**Table 2:** Inter-correlations between predictor variables and outcome variables.

Variables	ESE	ISE	SE-discrep	Sign of diff	Main diagn	Treat durat	Alc abuse	Suicid idea	BDI-1	BDI-2	BDI-3	BDI-4	BDI-5
ESE	1												
ISE	-0.09	1											
SE-discr	-0.07	0.14	1										
Sign of diff	0.64**	-0.58**	-0.23	1									
Main diagn	-0.51**	-0.12	0.01	-0.11	1								
Treat durat	-0.41*	-0.08	-0.05	-0.26	0.11	1							
Alc abuse	-0.09	0.29	0.20	-0.08	-0.05	-0.03	1						
Suicid idea	0.13	0.21	0.46**	-0.19	0.02	0.02	0.09	1					
BDI-1	-0.13	0.42*	0.38*	-0.25	0.32	-0.17	0.18	0.34	1				
BDI-2	-0.29	0.19	0.51**	-0.33	0.18	0.08	0.01	0.15	0.66**	1			
BDI-3	-0.24	0.33	0.45*	-0.37*	0.07	0.09	0.14	0.18	0.64**	0.89**	1		
BDI-4	-0.34	0.25	0.49**	-0.42	0.07	0.18	0.10	0.22	0.41**	0.69**	0.84**	1	
BDI-5	-0.14	0.21	0.42*	-0.26	0.14	-0.13	-0.10	0.15	0.36*	0.53**	0.66**	0.74**	1
BDI-6	0.09	0.06	0.33	0.05	0.13	-0.36*	-0.23	-0.11	0.38*	0.48**	0.47**	0.40*	0.65**

ESE = explicit self-esteem; ISE = implicit self-esteem; SE-discrep = self-esteem dicrepancy; Sign of diff = sign of self-esteem difference; Main diagn = main diagnosis; Treat durat = treatment duration; Alc abuse = alcohol abuse; Suicid idea = suicidal ideation; BDI = Beck Depression Inventory; sign of self-esteem difference is coded as -1 = higher ISE than ESE and 1 = higher ESE than ISE; main diagnosis is coded as -1 = major depressive disorder and 1 = recurrent depressive disorder; treatment duration in weeks; alcohol abuse is coded as -1 = no and 1 = yes; suicidal ideation is coded as -1 = no and 1 = yes;

*p < .05,

**p < .01.

Table 3 shows the results of the treatment-effect model. Concerning the diagnostic effects, self-esteem consistency is the only significant predictor of the three self-esteem measures. The positive regression coefficient indicates that the higher the self-esteem discrepancy was, the higher were the average BDI scores. The sign of difference between ESE and ISE was not important. Neither ESE nor ISE alone performed well as predictors of overall BDI scores. Alcohol abuse was the only other (control) variable that influenced the BDI scores in general, indicating that patients with a comorbid diagnosis of alcohol abuse had overall lower BDI scores.

**Table 3:** Treatment-effect model (BDI 1–5). Standardized regression coefficients from the two-level regression model predicting the diagnostic effects on overall BDI scores and the therapy-process effects.

Predictor	Regression coefficient β	Standard error	*t*	*p*
**Diagnostic effects**				
ESE	-0.02	0.21	-0.08	.937
ISE	0.16	0.16	1.05	.308
Self-esteem discrepancy	0.43	0.11	3.73	.001**
Sign of SE difference	-0.11	0.18	-0.62	.543
Gender	-0.20	0.13	-1.62	.120
Age	-0.001	0.11	-0.01	.994
Main diagnosis	0.05	0.11	0.49	.627
Treatment duration	-0.03	0.13	-0.21	.835
Alcohol abuse	-0.17	0.09	-1.86	.078*
Suicidal ideation	-0.06	0.15	-0.37	.718
**Therapy process**				
Point of measurement	-0.41	0.05	-7.70	< .001**
ESE	0.03	0.08	0.35	.731
ISE	-0.04	0.07	-0.60	.559
Self-esteem discrepancy	-0.09	0.04	-2.03	.056*
Sign of SE difference	-0.10	0.08	-1.19	.247
Gender	-0.10	0.07	-1.39	.181
Age	-0.001	0.05	-0.02	.988
Main diagnosis	-0.06	0.09	-0.65	.526
Treatment duration	-0.02	0.06	-0.34	.738
Alcohol abuse	-0.09	0.05	-1.89	.074*
Suicidal ideation	0.04	0.07	-0.62	.542

The *p* values are two-tailed; two stars mark *p* values below an alpha level of .05; one star marks *p* values below an alpha level of .10; *df* = 20 in each case; BDI = Beck Depression Inventory; ESE = explicit self-esteem; ISE = implicit self-esteem; SE = self-esteem; sign of self-esteem difference is coded as -1 = higher ISE than ESE and 1 = higher ESE than ISE; gender is coded as -1 = male and 1 = female; age in years; main diagnosis is coded as -1 = major depressive disorder and 1 = recurrent depressive disorder; treatment duration in weeks; alcohol abuse is coded as -1 = no and 1 = yes; suicidal ideation is coded as -1 = no and 1 = yes; main point of measurement is coded as 1 to 5 for BDI-1 to BDI-5.

Concerning the therapy-process effects, first, we obtained a significant negative regression coefficient for point of measurement, meaning that the depressive symptoms reduced over the course of therapy. Thus, the treatment was effective as intended. Of the three self-esteem measures, self-esteem discrepancy was the only significant predictor of the BDI-score reduction (at an alpha-level of .10 for two-tailed testing). This means that patients with high self-esteem consistency improved faster during the therapy. The sign of difference between ESE and ISE again did not play a role. Also, neither ESE nor ISE alone predicted the development of the BDI scores in the therapy process. Again, alcohol abuse was the only control variable that effected the therapy progress with a faster symptom reduction for patients without a comorbid diagnosis of alcohol abuse.

Table 4 displays the results of the treatment-stability model. Here, we found no significant predictor for the difference between BDI-6 and BDI-5. This contrasts with our expectations: Neither kind of self-esteem measure can predict treatment stability. Note, however, that the symptom reduction was relatively stable four months after the therapy. Thus, a significant predictor in this analysis would explain variance between the depressive symptoms at the end of the therapy and at the 4-months follow-up. Since there was not much variance, it is reasonable that no significant predictor could be found.

**Table 4:** Treatment-stability model. Standardized regression coefficients from the simultaneous regression model predicting the difference between BDI scores at the 4-month follow-up (BDI-6) and at the end of the therapy (BDI-5).

Predictor	Regression coefficient β	Standard error	*t*	*p*
ESE	0.17	0.40	0.42	.678
ISE	0.04	0.28	0.15	.883
Self-esteem discrepancy	0.19	0.22	0.86	.398
Sign of SE difference	0.21	0.40	0.53	.605
Gender	-0.12	0.24	-0.52	.611
Age	0.24	0.20	1.21	.241
Main diagnosis	0.12	0.24	0.48	.637
Treatment duration	-0.16	0.21	-0.77	.450
Alcohol abuse	-0.17	0.22	-0.77	.451
Suicidal ideation	-0.34	0.23	-1.49	.153

The *p* values are two-tailed; *df* = 23 in each case; BDI = Beck Depression Inventory; ESE = explicit self-esteem; ISE = implicit self-esteem; SE = self-esteem; sign of self-esteem difference is coded as -1 = higher ISE than ESE and 1 = higher ESE than ISE; gender is coded as -1 = male and 1 = female; age in years; main diagnosis is coded as -1 = major depressive disorder and 1 = recurrent depressive disorder; treatment duration in weeks; alcohol abuse is coded as -1 = no and 1 = yes; suicidal ideation is coded as -1 = no and 1 = yes.

**Fig 1:**
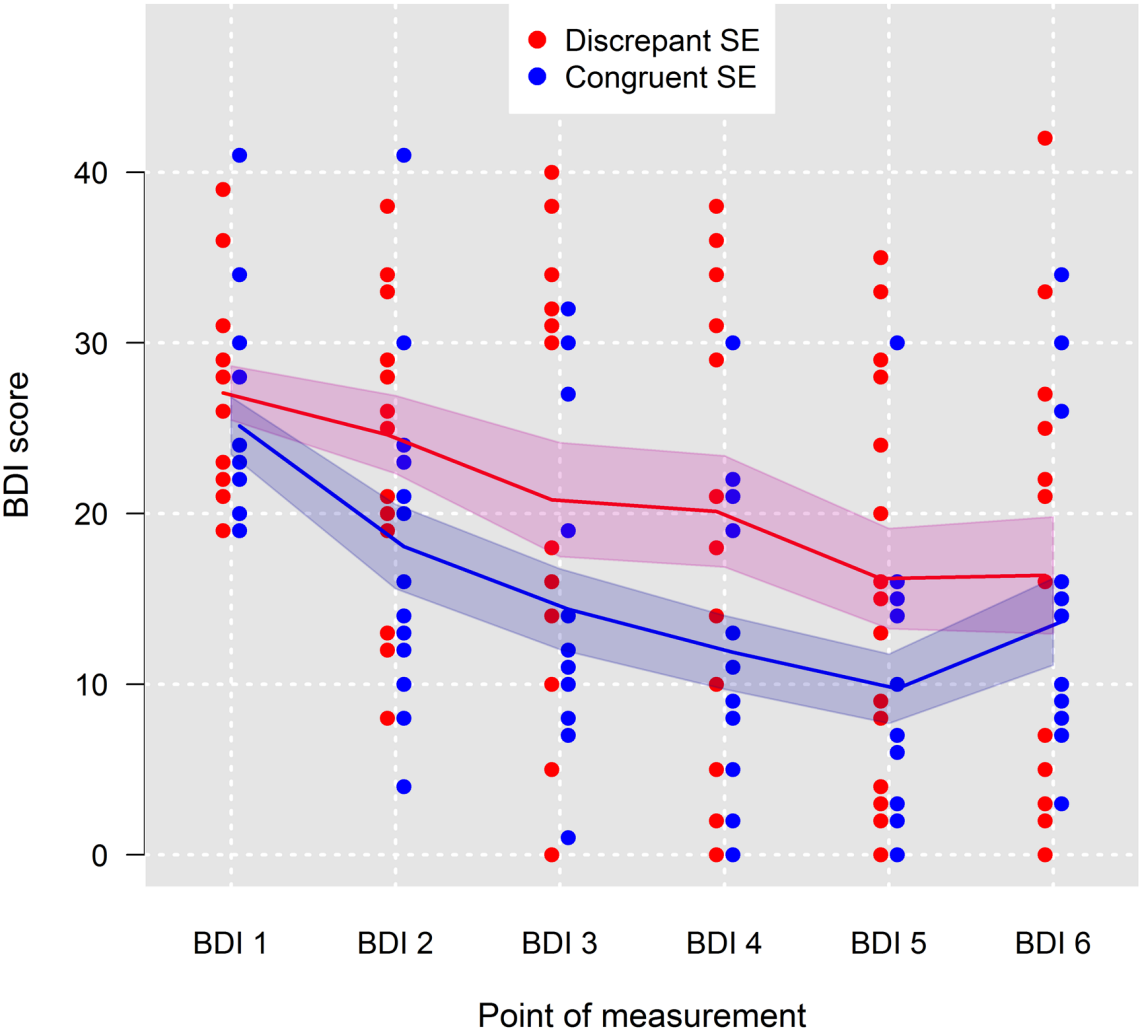
Fitted mean trends of Beck Depression Inventory (BDI) scores for patients grouped by means of a median split into patients with congruent self-esteem (*n* = 15) and patients with discrepant self-esteem (*n* = 16). Red and blue shaded areas are 95% confidence intervals; SE = self-esteem.

Fig 1 shows the BDI scores over all points of measurements (BDI-1 to BDI-6) of all patients. To allow for better visualization of the effect of self-esteem consistency on BDI scores, patients were divided by means of a median split for self-esteem discrepancy into groups with congruent (*n* = 15) and discrepant (*n* = 16) self-esteem. As can be seen in the figure, BDI-2 to BDI-5 scores of patients with congruent self-esteem were generally lower than those of patients with discrepant self-esteem. Also, BDI scores decreased slightly faster in patients with congruent self-esteem. These patients benefited more quickly from the treatment. At the 4-month follow-up, however, there was no difference in BDI scores between patients with congruent versus discrepant self-esteem. Fig 1 also shows a typical slight increase in BDI scores after the end of the treatment.

**Fig 2:**
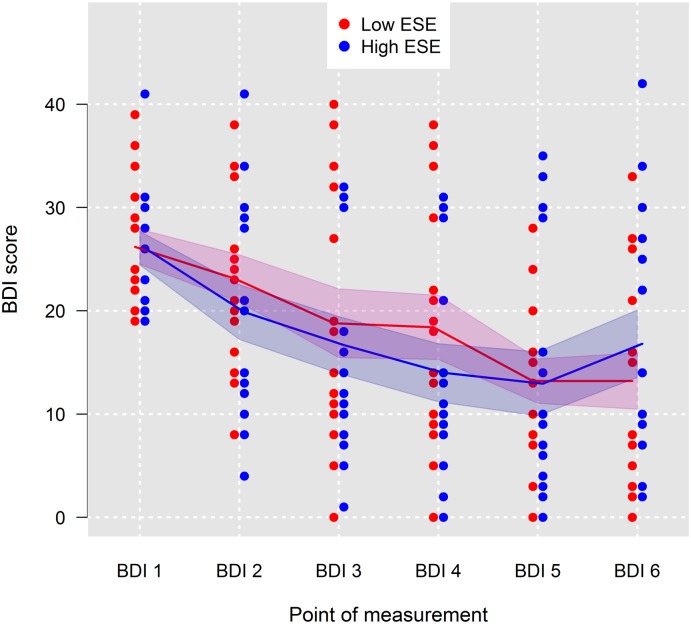
Fitted mean trends of Beck Depression Inventory (BDI) scores for patients grouped by means of a median split into patients with low explicit self-esteem (*n* = 15) and patients with high explicit self-esteem (*n* = 16). Red and blue shaded areas are 95% confidence intervals; ESE = explicit self-esteem.

Fig 2 displays all BDI scores (BDI-1 to BDI-6) for patients grouped by means of a median split for ESE into patients with high ESE (*n* = 16) and low ESE (*n* = 15). Here, no difference in the shape of the curve can be seen, neither concerning the degree of depressive symptoms nor regarding the progress in the course of the therapy.

**Fig 3:**
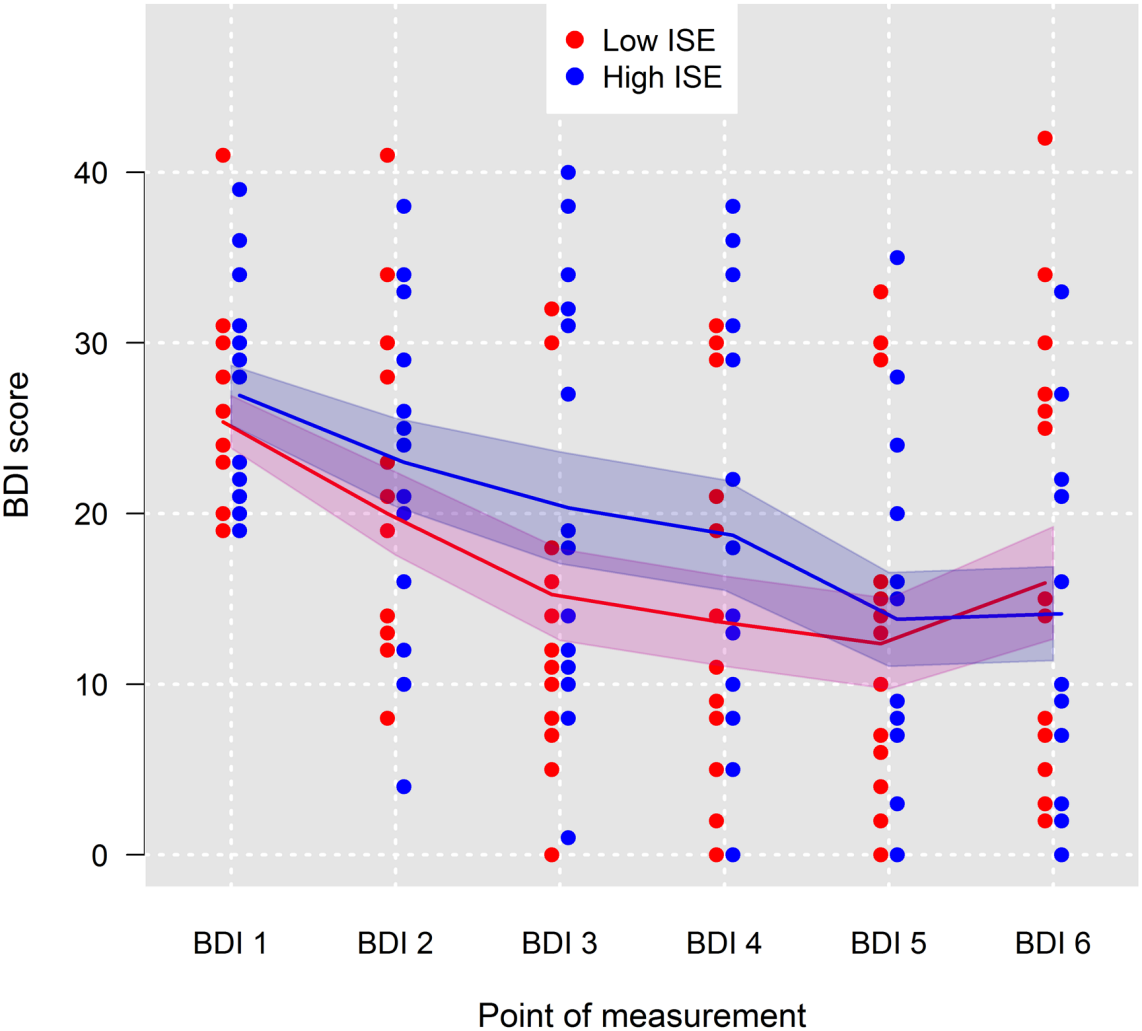
Fitted mean trends of Beck Depression Inventory (BDI) scores for patients grouped by means of a median split into patients with low implicit self-esteem (*n* = 16) and patients with high implicit self-esteem (*n* = 15). Red and blue shaded areas are 95% confidence intervals; ISE = implicit self-esteem.

BDI scores (BDI-1 to BDI-6) for patients grouped by means of a median split for ISE into patients with high ISE (*n* = 15) and low ISE (*n* = 16) are shown in Fig 3. Again, no difference in the shape of the curve occurred. The degree of depressive symptoms did not differ much between patients holding a high versus a low ISE. This also holds for the development of symptoms over the course of the therapy.

**Fig 4:**
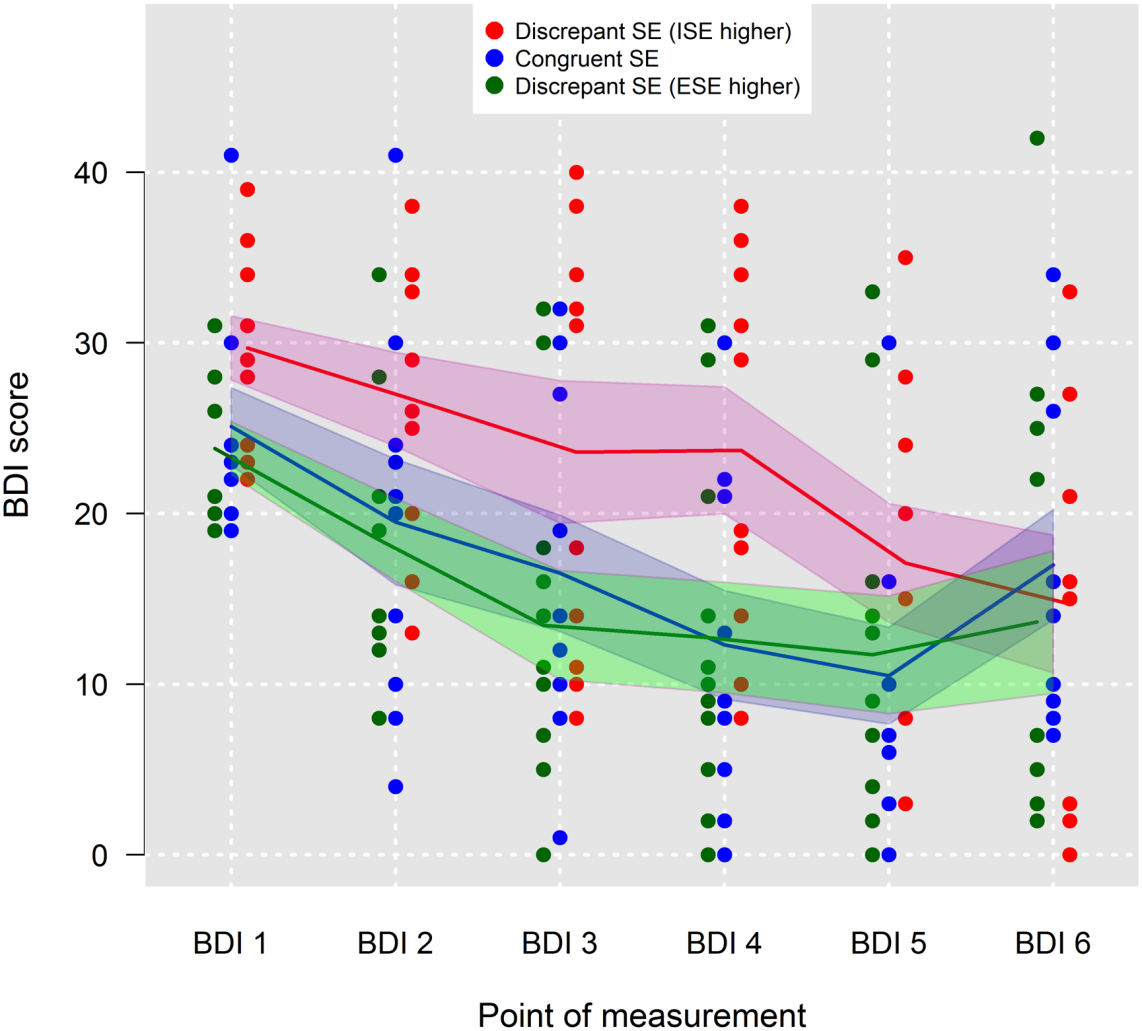
Fitted mean trends of Beck Depression Inventory (BDI) scores for patients grouped by means of 33.3% percentiles of the distribution of the difference between ESE and ISE into patients with congruent self-esteem (*n* = 10) and into patients holding one of two types of discrepant self-esteem, that is, higher implicit self-esteem than explicit-self-esteem (*n* = 11) and higher explicit self-esteem than implicit-self-esteem (*n* = 10). Red, blue and green shaded areas are 95% confidence intervals; SE = self-esteem.

Although in the multilevel model of treatment effects, the sign of self-esteem difference neither significantly predicted the average BDI scores nor the therapy progress, we performed a visual examination of the data to see whether there might be some trends. To do this, we divided the patients into three equally large groups (33.3% and 67.3% percentiles of the difference between ESE and ISE). Thus, we created a group of patients holding a congruent self-esteem (*n* = 10), that is, the one-third of patients in the middle of the distribution, and two groups of patients holding a discrepant self-esteem–the group of patients with a higher ISE than ESE (i.e., damaged self-esteem; *n* = 11) and the patients with higher ESE than ISE (i.e., fragile self-esteem; *n* = 10). Fig 4 indeed provides some additional tentative information. For the group of patients holding a damaged self-esteem, the first five BDI-scores were on average markedly higher than in the other two groups of patients. Also the symptom reduction in the course of the therapy seems to be slower than in the other two groups. However, no difference in the course of the curves was obtained between patients with a congruent self-esteem and a fragile self-esteem. Note, that the number of participants in each of the three groups was too low to produce statistical meaningful results. Thus, this visual examination can be just of explorative character.

## Discussion

In this study, the difference between ISE and ESE (self-esteem discrepancy) predicted depressive symptoms over the course of therapy. For discrepant self-esteem, depressive symptoms were more severe during the entire process of treatment. In addition, patients with discrepant self-esteem responded more slowly to treatment, yet the improvement reached finally was as stable as it was for patients with congruent self-esteem.

Concerning the relation between self-esteem consistency and severity of symptoms, for both forms of discrepant self-esteem, namely damaged self-esteem (i.e., higher ISE than ESE) and fragile self-esteem (i.e., higher ESE than ISE) disadvantageous effects are known from the literature. Damaged self-esteem has been associated with more anger suppression [24] and with depressive disorders (e.g., [28,45]). As pointed out by Rudman et al. [46], high ISE may be a compensatory reaction in case of threats to the explicit self-evaluation. Since damages to ESE are quite common in depression, high ISE may be a consequence. The stronger this compensatory reaction is, the more severe the damage to ESE may have been. This could explain why damaged self-esteem is related to more severe symptoms in depression. Groups of individuals with fragile self-esteem in turn have been discussed to show high scores of narcissism [23]. This suggests that mechanisms of self-presentation and self-overestimation compensate for low ISE. This in turn will make these individuals more likely to decompensate in response to threats to their explicit self-evaluation (e.g., failure, criticism, loss of social attention) because it diminishes their compensating strategy. More severe depressive symptoms may be the consequence of such experiences. However, some studies also highlight disadvantageous effects of congruent low self-esteem. A recent study by de Jong et al. [47] reported that congruent low self-esteem, rather than discrepant self-esteem, was correlated with higher social anxiety in girls. This is in line with Glashouwer et al. [48] who found that suicidal ideation was especially high for patients with low ESE and ISE. The inferential analysis of the current data revealed no pronounced differences concerning the direction of self-esteem discrepancy (i.e., damaged versus fragile self-esteem). However, a visual examination indicated that patients holding a damaged self-esteem might suffer from more severe depressive symptoms and show a slower symptom reduction over the course of the therapy. This is consistent with the results of Creemers et al. [28], who obtained increased levels of depressive symptoms in students holding a damaged self-esteem. In the current study, the statistical power of the multi-level regression analysis for examining the direction of the self-esteem difference may have been too low to reach statistical significance. Further research should be directed at clarifying the relationship between direction of self-esteem discrepancy and symptom severity.

How can one explain that discrepant self-esteem was related to slower treatment progress? A speculative explanation may be that discrepant self-esteem could mirror a low ability for self-reflection, which is an important factor for treatment progress and change. This idea is supported by Pelham et al. [49] who found less self-esteem discrepancy in women than in men. The authors explained this finding by women being more aware of their feelings. Cognitive behavioral therapy addresses mostly explicit thoughts and evaluations rather than implicit processes. When patients have a lower ability for self-reflection, it may be difficult and more time-consuming to influence implicit evaluations and spontaneous behavior, as they are not directly addressed in treatment and will not be as easily accessible to people with low abilities of self-reflection, compared to people with high abilities of self-reflection. This may explain why patients with discrepant self-esteem need more time to profit from therapy.

In contradiction to our hypotheses, we did not find any relation between self-esteem consistency and the stability of treatment effects. The reduction of depressive symptoms at the end of the therapy remained relatively stable up to four months after the therapy. Thus, because of the small variance in the dependent measure, it is reasonable that no significant predictors could be found.

In each of the analyses in the current study, we controlled for factors that could be expected to affect the severity of depressive symptoms and the therapy progress. Thus, we controlled for the impact of gender, age, main diagnosis, treatment duration, the comorbid diagnosis of alcohol abuse and suicidal ideation. This means, the result that self-esteem congruency is a significant predictor of the severity of depressive symptoms and the therapy progress cannot be explained by any of these variables. This is especially interesting in respect to suicidal ideation, since some studies found a relationship between suicidal ideation and self-esteem consistency. For example, Creemers et al. [28] showed that damaged self-esteem was associated with suicidal ideation in a student sample. Also Franck et al. [27] found a similar relationship. Depressed patients with suicidal ideation exhibited a damaged self-esteem while depressed patients without suicidal ideation held a congruent low self-esteem. In the current study, we also obtained a positive correlation between suicidal ideation and self-esteem discrepancy (see Table 2). However, the regression analyses that control for alternative explanations, show that suicidal ideation does not explain the severity of depressive symptoms and the symptom reduction over the course of the therapy.

Clearly, a limitation of this study is the rather small sample size. Even though we did find significant effects, the study’s power was not sufficient for the detection of smaller effects. This raises the need to replicate the study with a larger sample size. A large-scale replication would also allow for more thorough separate analyses of the two types of discrepant self-esteem (fragile and damaged) and the two types of congruent self-esteem (low and high).

It might also be advantageous to add a control group of a comparable non-depressed sample. This would make it possible to examine whether there is a difference in ISE, ESE and self-esteem congruency already at baseline. Although previous studies compared the relationship between the three self-esteem measures and the degree of depressive symptoms between healthy controls and different clinical samples of depressed patients, their results differ. Franck et al. [27] found higher ISE, ESE and self-esteem congruency in non-depressed controls. However, in the study of van Tuijl et al. [29], the non-clinical sample only systematical differed regarding ESE, with a higher ESE in the control group compared to all clinical groups.

A further limitation of the current study is the individualized therapy the patients received. All patients received a partially standardized program of depression treatment; however, they were also individually treated in accordance with their individual needs. Thus, we could not control for the content of individualized single therapy sessions. Additionally, the duration of the therapy varied between four to eight weeks depending on the patients’ therapy progress. The advantage of this procedure is that an individualized treatment is common practice, giving our study higher external validity.

The affective priming task [36] that was modified and used in this study has previously been criticized for rather low internal consistencies and low temporal stability coefficients [11]. In response, interesting variations of the priming task were introduced. Krause et al. [50] presented individualized pictures of the participants as self-relevant primes, rather than words. Wentura et al. [51] prevented high levels of response accuracy by presenting a modification of the affective priming task, such that participants are forced to respond to the targets within a limited time span. Both variations of the affective priming task improved the reliability of the original affective priming task by Hetts et al. [36]. These adjustments of the task should be considered in future studies to improve reliability of the affective priming task.

A general problem that probably all studies on implicit self-esteem face, concerns the way ISE is measured. As yet, whether a patient is high or low in ISE is always determined in relation to other members of the sample. When addressing inconsistency of self-esteem, this naturally implies the use of the difference of standardized measures of ISE and ESE for prediction rather than absolute values of congruent and discrepant self-esteem, thus creating sample-dependent results. It is still an open question how discrepant self-esteem would influence depressive disorders if it was possible to identify patients with high versus low ISE on a population based norm, as is the case for ESE data.

Given the external validity and some clear-cut findings of this study, namely that depressed patients with congruent self-esteem improved faster than those with discrepant self-esteem, practical implications can be derived. Most importantly at that is to answer the open questions whether discrepant self-esteem could be directly targeted by therapy and whether this in itself would improve treatment outcome, and might even reduce the risk of relapse. In case of low ESE, explicit self-enhancement may be especially important to target. This is typically done in cognitive behavioral therapy. Dijksterhuis [52], on the other side, showed promising effects of enhancing ISE, which may help patients suffering from low ISE. Attempts to change maladaptive cognitive biases by cognitive bias modification programs (CBM) have been successfully made in the field of anxiety disorders and, to some extent, substance use disorders. Yet, CBM programs have rarely been implemented in treatment-as-usual protocols, assessing their practical value. Adding trainings of implicit processes to treatment that is usually focused on explicit processes may improve the current therapy practices.

Contrary to our expectations, we did not find any differences regarding the stability of treatment effects between patients differing in their self-esteem consistency. As already discussed, the reason for the lack of difference could be a stable therapy success for the majority of the patients–at least within the follow-up period of four months. Alternatively, the lack of relation between self-esteem consistency and treatment stability could be explained by the option that the consistency of ESE and ISE does not matter any longer once a successful therapy of any kind was implemented. Although, it is certainly essential to offer a therapy that suits the particular type of self-esteem combination (because this will foster a stronger and faster symptom reduction), however, if the abovementioned is right, no concerns have to be raised regarding a more probable or faster relapse in the case of an initially higher self-esteem discrepancy once the therapy was successful. In other words, self-esteem discrepancy does not necessarily lead to weaker therapy success; it just means that the right and most efficient kind of therapy for each type of self-esteem combination has to be developed.

## Supporting information

S1 Data Dataset. (XLSX)
